# Measurable residual disease testing and allogeneic hematopoietic cell transplantation for AML: adapting Pre-MEASURE to clinical practice

**DOI:** 10.1038/s41409-024-02481-2

**Published:** 2024-11-20

**Authors:** Brian C. Shaffer, Partow Kebriaei, Marcos de Lima, Antonio M. Jimenez Jimenez

**Affiliations:** 1 Adult Bone Marrow Transplantation Service, Department of Medicine, Memorial Sloan Kettering Cancer Center, New York, NY USA; 2 Department of Stem Cell Transplantation and Cellular Therapy, Division of Cancer Medicine, The University of Texas MD Anderson Cancer Center, Houston, TX USA; 3 Division of Hematology and Oncology, and Stem Cell Transplant Program, Case Western Reserve University, Cleveland, OH USA; 4 Division of Transplantation and Cell Therapy, Sylvester Comprehensive Cancer Center, Miller School of Medicine, University of Miami, Miami, FL USA

**Keywords:** Leukaemia, Acute myeloid leukaemia

## Abstract

Measurable residual disease (MRD) testing in patients with acute myelogenous leukemia (AML) represents a heterogenous assessment process designed to quantify leukemia-specific biomarkers that are not ascertainable by routine pathologic evaluation. The most common tools used to assess MRD are multiparameter flow cytometry (MPFC), and polymerase chain reaction (PCR) based tools, including quantitative or digital droplet PCR (qPCR, ddPCR), or next-generation sequencing (NGS) technologies. Collectively, MRD assessments have become an important clinical tool in the management of patients with AML. Despite progress, significant questions remain with respect to the appropriate timing, frequency, and methodology of MRD assessment, and whether or how to adapt therapy based on MRD results. Recent data from the Pre-MEASURE study, a retrospective cohort analysis of error corrected NGS based MRD assessment prior to allogeneic hematopoietic cell transplantation (alloHCT) in patients with AML, provides additional key information with respect to the emerging role of NGS-based technology in MRD assessment. In the context of this review, we evaluate the Pre-MEASURE study as well as other recent, high-quality assessments of MRD in AML. Our focus is to provide a practical assessment of the use of emerging MRD technologies in patients with AML with an emphasis on the role of peri-transplant MRD for the practicing clinician.

## Introduction

Acute myeloid leukemia (AML) is a genetically complex and heterogeneous disease arising from the clonal proliferation of hematopoietic lineage-derived cells [1, 2]. AML is relatively rare but is one of the most common types of leukemia in adults, occurring in 4.2 individuals per 100,000, and increases in incidence with age [3]. Despite advances in care, <40% of patients diagnosed with AML will survive beyond 5-years from diagnosis, reflecting the aggressive nature of the disease and limitations of current therapy. The process of leukemogenesis is likely multifactorial and complex, involving disordered epigenetic regulation, cell proliferation, and microenvironmental interaction [4]. Leukemic transformation typically involves the acquisition of mutations in oncogenes and tumor suppressor genes, resulting in the display of an abnormal cell surface protein repertoire known as the leukemia-associated immunophenotype (LAIP), which is distinct from that of healthy hematopoietic tissue [5–7]. The diagnosis of AML thus depends on routine pathologic review, cytogenetic analysis via karyotyping or fluorescent in-situ hybridization approaches, and sequencing of specific driver oncogenes. Relapse is common in patients who achieve an initial remission, reflecting the limitations of conventional pathologic tools to identify small amounts of residual AML [8].

The notion of measurable residual disease (MRD) assessment, the ability to distinguish AML cells at a lower resolution that can be achieved with conventional pathological tools (typically <10^−2^ ratio of abnormal cells to total leukocytes analyzed or abnormal allele ratio, AR), is approximately 40 years old [9]. MRD assessment has evolved with emergent technologies, including the use of multiparameter flow cytometry (MPFC) to identify and distinguish LAIP, sensitive quantitative or digital droplet polymerase chain reaction (qPCR, ddPCR) assessments, and more recently the use of next-generation sequencing (NGS) technologies with error-correction to identify AML associated gene mutations at low AR. Contemporary approaches to MRD assessment can identify AML involvement in an AR resolution of ~10^−4^–10^-5^, a major step forward in resolution [10, 11]. MRD assessments now offer the opportunity to refine available treatments based on an enhanced appraisal of disease response.

The Pre-MEASURE study was a retrospective evaluation of error-corrected NGS-based MRD assessment in patients with AML prior to allo HCT [12]. Patients were treated at multiple centers and MRD assessment was centralized. The aim of the study was to determine whether a sensitive and well-standardized approach may be applicable in a large, diverse, AML population and to compare with individual center-based MRD assessments. The purpose of this review is to identify the advents in technology that led up to the Pre-MEASURE study, review what new knowledge was gained from this work, and look forward to what information may help to improve AML treatment in the future. We approach this from the perspective of a practicing clinician, with a focus on MRD to inform the optimal timing of alloHCT in AML.

### Multi-parameter flow cytometry, next-generation sequencing, and error-correction technologies in quantifying measurable residual disease

MPFC, qPCR, and NGS are the primary MRD assessment techniques in AML are summarized in Table 1. Each has unique advantages and limitations:

**Table 1:** Major MRD Assessment Technologies.

Method	Sensitivity	Markers	Advantages	Applicability	Disadvantages
Quantitative Polymerase Chain Reaction (qPCR)	10^−4^ to 10^−5^ (up to 10^−6^ with adequate material)	*NPM1, RUNX1-RUNX1T1, CBFB-MYH11, PML-RARA, KMT2A-MLLT3, DEK-NUP214, BCR-ABL*, and *WT1*.	- Highly sensitive and specific -Well-standardized platform - Effective for specific fusion transcripts and mutations	~50%	- Limited to subsets of AML patients with specific mutations - Less than half of adult AML cases applicable
Next-Generation Sequencing (NGS)	10^−2^	*FLT3, IDH1, IDH2, NPM1*, *CEBPA*, *DDX41, TP53*, *ASXL1*, *BCOR*, *EZH2*, *RUNX1*, *SF3B1*, *SRSF2*, *STAG2*, *U2AF1*, *ZRSR2* and any mutation within sequenced regions^a^	-Comprehensive assessment of multiple genes - Detects any mutation within sequenced regions - Potential for non-invasive testing with circulating cell-free DNA	~90%	Higher intrinsic error rates result in low sensitivity May detect gene mutations not associated with leukemogenesis (DTA genes)
Error Corrected NGS	10^−3^ to 10^−4^	Same	Improved sensitivity compared to standard NGS technology.	~90%	Higher cost, sequencing instrument use

^a^Due to their non-informative nature for MRD, germline mutations in *ANKRD26*, *CEBPA*, *DDX41*, *ETV6*, *GATA2*, *RUNX1*, and *TP53* should be excluded as NGS-MRD markers. DTA (*DNMT3A*, *TET2*, *ASXL1*) mutations should also be excluded, as they are commonly found in age-related clonal hematopoiesis and do not represent the leukemic clone.

MPFC uses fluorochrome-conjugated antibodies that recognize abnormal proteins on leukemic blasts to discern leukemic blasts from healthy tissue [6, 13]. Because there is significant heterogeneity in phenotypic patterns between leukemia cases, MPFC employs a “different-from-normal” strategy to resolve MRD [14]. When conducted by experienced laboratory and pathology scientists, MPFC represents a test that has a relatively short turnaround time, is widely available, and can achieve a detection limit of 10^-4^. The main limitation of this technology is lack of standardization, resulting in inter-laboratory variation that can be difficult to adapt to multicenter inquiry [15].

qPCR-based methods, in general, offer similar to greater resolution compared to MPFC with the potential for greater standardization [16, 17]. qPCR achieves sensitivities of 10^−4^ to 10^−5^, with the potential for 10^−6^ sensitivity with sufficient genetic material [18–20]. Digital droplet PCR (ddPCR) is an advanced methodology that employs the use of water-oil emulsion to facilitate the PCR reaction [21]. ddPCR demonstrates greater resilience against contaminants and inhibitors, enhancing its reliability in MRD assessment [22]. Use of both qPCR and ddPCR are restricted to well-validated gene mutations or rearrangements: The ELN recommends qPCR for initial MRD assessment in AML patients with *NPM1*, t(8; 21), inv [16], and t(15;17). Thus, while both qPCR and ddPCR technologies are reproducible and sensitive, they are limited by their restriction to specific genetic aberrations [10].

Next-generation sequencing represents a heterogenous approach designed to use massive, short-read sequencing of genomic DNA or complementary DNA libraries (gDNA, cDNA), followed by bioinformatic resolution and quantification of abnormal gene sequences associated with leukemia [2, 5]. Sequencing errors in NGS technologies typically occur at a rate of 10^-2^-10^-3^, limiting the practical sensitivity of standard NGS to about 1–2% of variant reads when used on nucleic acid material derived from admixed tissue [23–25]. Targeted NGS is essential for disease classification and prognosis and is used for the serial evaluation of mutations identified at diagnosis. The ELN recommends NGS screening with a multigene panel that considers all detected mutations as potential MRD markers [26, 27]. However, caution is necessary when analyzing germline and age-related mutations associated with clonal hematopoiesis (Table 1) [28–30]. Given these technological limitations, the ELN does not yet recommend NGS as a stand-alone technique in routine MRD assessment due to a lack of standardization and incomplete validation of target mutations.

More recently, the use of error-correction-based NGS approaches demonstrated improved resolution over standard NGS [24, 31]. With error-correction, a unique molecular identifier (UMI) is applied early during cDNA library generation to allow for the bioinformatic removal of mutations that are introduced during sequence amplification. While error correction reduces the detection limit of NGS to ~10^−4^, the application of this technology is currently limited by cost and sequencing instrument resource availability [32].

### Major findings from the Pre-MEASURE study

The Pre-MEASURE study represents an important step in evaluating the prognostic impact of pre-HCT NGS-MRD on post-HCT outcomes [12]. Here, blood samples from 1075 adult AML patients were subjected to error-corrected NGS-based MRD assessment using a gene panel that included *NPM1, FLT3-ITD, IDH1, IDH2*, and *Kit*. The primary finding from the study was that patients with residual *NPM1* and/or *FLT3*-ITD variants prior to allo HCT (AR > 0.01%) had higher relapse rates (68% vs. 21%; *P* < 0.001) and poorer 3-year survival (39% vs. 63%; *P* < 0.001) compared to MRD^-^ patients. A second important finding was that center-based MPFC did not correlate in a significant degree with core clinical outcomes. Finally, the adverse impact of pre-HCT MRD was shown to be partially mitigated in younger patients who received high-intensity MAC (3-year relapse rate, 53% vs. 78% for RIC; *P* = 0.04; 3-year OS, 42% vs. 33% for RIC; *P* < 0.001), suggesting that more aggressive conditioning regimens could offset some of the adverse effects of MRD positivity at transplant. Exploratory analyses revealed that NGS-MRD^+^ patients who received melphalan-based RIC experienced significantly lower relapse rates and improved survival.

In another cohort study by the same research team, the dose-dependent relationship between pretransplant MRD levels, as measured by NGS detection of *FLT3*-ITD, and post-transplant outcomes was further confirmed [33]. Patients receiving RIC without melphalan or nonmyeloablative (NMA) conditioning exhibited an increased risk of relapse and death compared to those receiving RIC with melphalan or MAC, underlining the crucial role of tailored conditioning strategies based on MRD status.

Further supporting the utility of NGS MRD, Dillon et al. compared MRD detection in 62 AML patients in first complete remission from the SWOG-S0106 trial using duplex sequencing (DS), a highly sensitive DNA sequencing method that identifies rare mutations by error-correcting both DNA strands, significantly reducing sequencing errors [34]. This technique involves shearing genomic DNA, followed by end repair, adapter ligation, and multiple rounds of hybridization and PCR before sequencing, allowing for the precise detection of mutations at extremely low variant allele frequencies. DS identified MRD in 35% of patients, strongly correlating with higher relapse rates and lower 5-year survival compared to MPFC, which detected MRD in only 16% of patients. This highlights the enhanced sensitivity and potential of DS NGS in refining prognostic assessments and informing treatment decisions.

The collective results of these retrospective cohort studies pave the way for the ongoing MEASURE study—a prospective trial to further validate MRD testing in patients undergoing transplantation, extending beyond *FLT3*-ITD and *NPM1* mutations.

### Pre-MEASURE in context: applying current knowledge to clinical practice

Major questions with respect to the use of MRD as a clinical decision tool in AML are addressed below: (1) Can MRD be used to defer allogeneic HCT in some patients? (2) Should MRD^+^ patients following induction receive additional therapy before going to allo HCT? (3) Should HCT conditioning therapy be adapted based on MRD testing? At the end of this section, we offer summarized recommendations based on how we incorporate the Pre-MEASURE and other key data into clinical practice.

### Can HCT be deferred in MRD-negative patients in the first CR?

Despite improvement in resolution via the use of error-corrected NGS, an overall relapse rate of >20% in MRD^-^ patients in Pre-MEASURE suggests that a nuanced approach to this population is required when appraising the value of HCT. Results from the GIMEMA AML1310 trial and the CETLAM-12 study provide additional context on using MRD to guide HCT strategies in newly diagnosed AML [35–37]. The GIMEMA trial employed a risk-adapted, MRD-directed approach, recommending allo HCT for intermediate-risk patients with detectable MRD after consolidation, while MRD^-^ patients received autologous HCT. This strategy resulted in closely comparable 2-year overall survival (79% for MRD^-^ vs. 70% for MRD^+^ patients receiving allo HCT; *P* = 0.713) and disease-free survival (61% for MRD^-^ vs. 67% for MRD^+^; *P* = 0.773) rates between the two groups, highlighting that personalized treatment based on MRD status can effectively manage patient outcomes without compromising survival. Similar findings were noted in an international study of 76 untreated *NPM1*-mutated AML patients treated with venetoclax and hypomethylating agent (HMA) or low-dose cytarabine (LDAC). MRD^-^ patients had an 84% 2-year OS, compared to 46% for MRD^+^ patients. Importantly, 22 patients who stopped therapy in MRD^-^ remission had an 88% 2-year treatment-free remission [38].

Expanding on this, the CETLAM-12 study illustrates explicitly the benefits of a preemptive approach to allo HCT in patients who show early signs of relapse or inadequate MRD clearance post-consolidation. In this protocol, patients with confirmed molecular failure (MF) or overt hematological relapse, indicated by MRD assessments, underwent allo HCT. This preemptive strategy led to notably favorable outcomes, particularly highlighted by the two-year overall survival rates being significantly higher in patients treated preemptively for MF than those who experienced relapse. This study also showed that for patients not presenting MF or hematologic relapse (MRD^-^), a watchful waiting approach coupled with rigorous MRD monitoring can be used to tailor allo HCT use. The group of patients who maintained sustained molecular remission experienced excellent survival outcomes, with a two-year overall survival rate of 91.3%.

Recent findings, such as those from the UK National Cancer Research Institute AML17 and AML19 trials, provide critical insights that challenge the conventional approach of frequently recommending immediate HCT for all patients with coexisting *NPM1* and *FLT3*-ITD mutations [39]. This study specifically investigated whether MRD could refine the selection of *NPM1*-mutated patients for allo HCT. The results revealed that patients with MRD present after two cycles of intensive chemotherapy benefited from higher overall survival rates following allo-HCT compared to those with undetectable MRD, who did not show the same benefit despite a higher risk of relapse if not transplanted in CR1.

These studies indicate that in some MRD-negative patients, deferring immediate transplantation while maintaining close surveillance can effectively minimize unnecessary exposure to HCT-related risks, particularly for frail or unfit patients. However, it is crucial to recognize that for patients with very high-risk genotypes (e.g., complex or monosomal karyotype, 17p/*TP53* mutations), achieving MRD negativity remains challenging and is still associated with high relapse rates, making a watchful waiting approach potentially inappropriate for these individuals [40, 41].

### Should patients with persistent MRD after induction therapy be offered additional treatment *versus* immediate HCT?

The timing of allo HCT in patients with persistent MRD after induction therapy for AML presents a critical decision point in treatment planning. While MRD positivity pre-HCT is associated with increased relapse rates and worse survival, it is unclear whether patients truly benefit from further, MRD-directed consolidative therapy due to lack of efficacy and risk of relapse prior to HCT. Thus, the optimal strategy of whether to proceed directly to HCT in MRD^+^ patients *versus* administering further therapy designed to eradicate MRD is unclear [42–45]. Studies that address this question are reviewed below.

A recent secondary analysis of the VIALE-A study reported by Pratz and colleagues is illustrative: Here, older, or unfit patients with AML were randomized to receive azacytidine with placebo or venetoclax 400 mg/day x 28 days per cycle [46, 47]. MRD was assessed serially by MPFC. Among 164 evaluable patients, 67 (41%) achieved an MRD^-^ CR (defined as <10^−3^). Importantly, only 17/67 patients that reached MRD^-^ CR did so by the first cycle, and 14/67 patients converted to MRD^-^ CR after cycle 7 of therapy, suggesting that prolonged therapy with a hypomethylating agent (HMA) can improve MRD response. Patients with higher-risk genomic mutations, such as in *TP53*, were less likely to achieve an MRD^-^ state.

Stahl et al. describe outcomes from a single-center study of patients undergoing chemotherapy-based induction. This treatment led to MRD-negative remission in 35% of patients, MRD-positive remission in 27%, and persistent disease in 38%. Following additional therapy, 34% of those with MRD-positive status and 26% of patients with persistent disease converted to MRD-negative status [48]. Patients with mutations in *CEBPA, NRAS, KRAS*, and *NPM1* were more likely to achieve MRD^-^ state; whereas those with mutations in *TP53, SF3B1, ASXL1*, *RUNX1*, and high-risk karyotype abnormalities were more likely to have sustained MRD. These data are further supported by work from Murdock and colleagues, who analyzed 295 older patients with AML who underwent non-intensive conditioning-based HCT [49]. While sequencing-based MRD assessment was associated with leukemia-free survival on univariable analysis, after adjusting for underlying disease risk parameters this association was not significant. Collectively these data suggest that analysis of MRD is confounded by underlying disease risk, higher-risk subgroups are unlikely to achieve an MRD^-^ state, and that specific subgroups such as *NPM1* and *IDH* mutated AML may benefit the most from MRD-directed treatment.

In patients with non-high-risk genomic or karyotype findings, it is unclear whether patients should proceed directly to HCT *versus* undergo additional treatment directed at eradicating MRD. The main risks of offering MRD-directed therapy prior to HCT are relapse and complications of treatment that may limit access to HCT. Some circumferential data exists in the form of benefit from combinatorial approaches to induction. For example, the addition of midostaurin to chemotherapy improved survival in patients who underwent subsequent HCT, suggesting that deeper remissions may improve subsequent HCT results [50]. Randomized trials in this space are urgently required.

In the interim, HCT appears to be beneficial in patients with MRD: The Acute Leukemia French Association Group provided compelling evidence that for adult patients with *NPM1*-mutant AML, allo HCT in first CR significantly improved both disease-free and overall survival for those with MRD (defined as less than a 4-log reduction following induction), compared to those who did not undergo allo-HCT [51]. This study also highlighted that for MRD^-^ patients or those achieving deep molecular responses before allo-HCT, the transplant did not yield additional survival benefits, emphasizing the critical role of MRD status in guiding treatment decisions. The Dutch-Belgian Hemato-Oncology Cooperative Group and Swiss Group for Clinical Cancer Research studied 1511 AML patients to assess the impact of MRD on post-remission treatments, including allo HCT, chemotherapy, or autologous HCT [52]. They found that MRD^+^ patients who achieved CR1 had significantly worse overall survival (50% vs. 65%, *P* < 0.001) and relapse-free survival (38% vs. 58%, *P* < 0.001) compared to MRD^-^ patients, mainly due to a higher relapse rate (54% vs. 32%, *P* < 0.001). Multivariable analysis further revealed that allo HCT markedly reduced the relapse incidence compared to other treatments for both MRD^+^ (HR, 0.35; *P* < 0.001) and MRD^-^ patients (HR, 0.38; *P* < 0.001), underscoring its efficacy regardless of MRD status.

Collectively, these results suggest the following: (1) Patients with high-risk genomic aberrations have a low probability of reaching an MRD^-^ state and a high risk of relapse; suggesting that allo HCT should not be deferred if the patient reaches an initial CR. (2) Some patient populations may benefit from additional therapy to eradicate MRD: Those with lower risk genomic mutations have a greater likelihood of conversion to an MRD^-^ state. Importantly, the high incidence of relapse in *NPM1* mutated patients with persistent MRD at allo HCT in Pre-MEASURE suggests that these patients may benefit from further treatment designed to eradicate MRD prior to HCT. Patients who are not fit to tolerate intensive conditioning may benefit from additional MRD-directed therapy; however, a well-defined standard of care is currently lacking. A large, genomically annotated prospective study is needed to address this situation.

### Should conditioning therapy be tailored to MRD assessment results?

Despite the known impact of pre-transplant MRD in allo HCT outcomes, evidence from several trials suggests variability in outcomes based on conditioning intensity (Table 2). For instance, Walter et al. found that MRD+ status prior to allo HCT, assessed using 10-color MFC, led to significantly worse three-year overall survival and relapse rates compared to MRD^-^ status [53]. Notably, while MRD^-^ patients had better outcomes with myeloablative conditioning (MAC), MRD+ patients experienced poor outcomes irrespective of the conditioning intensity, highlighting the critical importance of achieving MRD negativity prior to transplantation.

**Table 2:** Outcomes of selected studies evaluated DNA sequencing-based or multiparameter flow cytometry-based MRD assessments.

Study	Patient population	*N*	MRD Methodology	Major findings	Reference
**PCR OR NGS BASED**					
Pre-MEASURE	Adult patients in first remission with *FLT3*-ITD, *NPM1, IDH1*, *IDH2*, or Kit mutations	822	Error corrected PCR	3-year relapse in *FLT3*-ITD or *NPM1*+ patients 68% vs. 21%	[12]
Multicenter NGS	Adult patients >= 60 in CR1	295	Standard NGS	No association between MRD and outcomes in multivariable analysis; MRD+ was higher in patients with high-risk genomic aberrations.	[49]
AML17/AML19	*NPM1*-mutated AML	737	qPCR	MRD+ patients benefit from allo (3-year OS 61% vs. 24%); no benefit for allo in CR1-MRD- patients	[39]
BMT-CTN 0901	MAVRIC study participants; adult patients with AML	190	Error corrected PCR	MAC results in lower 3-year relapse (19% vs. 67%) and better survival (61% vs. 43%) in patients with any detected mutation	[45]
HOVON	Adult patients with *FLT3*-ITD	161	NGS	MAC results in improved outcomes in MRD+ patients	[59]
**Multiparameter flow cytometry-based**					
Seattle Cohort	Adult AML in CR	279	Different-from-normal	3-year OS: 75% in MRD-, 27% MRD+ 3-year relapse: 23% MRD-; 69% MRD+	[69]
IRCCS	Adult AML in CR	224	Different-from-normal Paired with WT1 NGS-based assessment	Cumulative relapse: 30% in MRD+ vs. 8.3% in MRD- 3-year OS: 73.5% in MRD- vs. 60% in MRD+ with WT1-negative and 36.7% in MFC+/WT1+	[70]
NCRI AML17	Younger adult patients	2450	Different-from-normal	HCT improved OS in MRD+ standard-risk patients (HR: 0.72) whereas no benefit for MRD-	[71]

The NCRI FIGARO trial explored the efficacy of an intensified RIC regimen, FLAMSA-Bu, against a standard fludarabine-based RIC regimen in older adults with high-risk AML or MDS [54]. This trial involved 244 patients and revealed no significant improvement in 2-year overall survival or cumulative incidence of relapse between the two regimens. Regardless of conditioning intensity, detectable pretransplant MRD was linked to higher relapse rates. Achieving complete donor T-cell chimerism at three months helped mitigate the adverse effects of pretransplant MRD, but the FLAMSA-Bu regimen did not enhance transplant outcomes over standard RIC.

Similarly, the NCRI AML 17 trial analyzed pretransplant blood and bone marrow samples from 107 patients with *NPM1*-mutant AML undergoing allo HCT [55]. Patients with different levels of MRD had varying 2-year overall survival rates—83% for MRD^-^, 63% for low-level MRD, and only 13% for high-level MRD (*P* < 0.0001). Among MRD+ patients, there was no significant correlation between overall survival and conditioning intensity, with 2-year overall survival rates of 50% for MAC versus 43% for RIC (HR, 1.22; CI, 0.54–2.76; *P* = 0.6). T-cell depletion was linked to significantly reduced survival across the entire cohort and particularly among MRD+ patients, emphasizing the nuanced effects of immune factors on post-transplant outcomes.

In stark contrast and supporting the benefits of conditioning intensification, the US CTN 0901 trial demonstrated a survival benefit for intensive conditioning in patients with myeloid neoplasia undergoing HCT [56, 57]. The prognostic value of pre-HCT MRD in AML was analyzed in a secondary analysis [45]. An error-corrected NGS panel was used to detect mutations in 13 commonly mutated AML genes in preconditioning blood samples. Among patients with any detectable mutation, those who received MAC had significantly lower relapse rates (19% vs. 67%) and higher three-year overall survival (61% vs. 43%) than those undergoing RIC. Multivariable analysis confirmed that RIC was associated with increased relapse, decreased relapse-free survival, and decreased overall survival in MRD^+^ patients based on this methodology.

A retrospective study by the European Society for Blood and Marrow Transplantation registry also supported the notion that MAC should be preferentially considered for young patients with pre-HCT MRD [58]. The study showed that non-intensive conditioning resulted in a higher risk of relapse and inferior leukemia-free survival compared to MAC. Further supporting the benefits of conditioning intensification, the recent PRE-MEASURE trial emphasized that young, MRD+ patients experienced improved post-transplant outcomes when undergoing conditioning intensification [12]. A recent analysis from the HOVON and SAKK clinical trials (HO42A, HO102, HO132 AML) demonstrated that MAC reduced relapse rates and improved overall survival in *FLT3*-ITD MRD AML patients compared to those receiving RIC, with similar non-relapse mortality rates between groups [59].

### Can peri-HCT MRD inform post-HCT maintenance strategies?

Suggested guidelines recommend *FLT3* kinase inhibitors as post-HCT maintenance for *FLT3*-ITD AML to reduce relapse risk, based on earlier randomized trials [60–62]. These trials were conducted before the standard use of *FLT3* inhibitors in primary leukemia therapy, leading to concerns about their current relevance. The MORPHO trial evaluated the use of post-HCT maintenance with gilteritinib, a selective *FLT3* kinase inhibitor, in adults with *FLT3*-ITD AML in first remission [63]. Participants were randomized to receive either gilteritinib or placebo for 24 months post-HCT. There was no statistically significant difference in the primary endpoint of relapse-free survival (HR, 0.679; *P* = 0.0518); however, a prespecified analysis demonstrated that patients with detectable MRD pre- or post-HCT had improved relapse-free survival with the use of gilteritinib (HR, 0.515; *P* = 0.0065), while those without detectable MRD did not [63, 64]. Gilteritinib maintenance may offer specific advantages for patients with MRD, particularly those with detected MRD following HCT. A salient question arising from this study is whether MRD detection post-HCT could be used to guide pre-emptive treatment, sparing some patients the cost and toxicity of maintenance therapy. This approach may logically extend to other inhibitors of leukemia-driving oncogenes, such as *IDH1/*2, menin, and others, but currently lacks prospective data [65–68].

Data from the ongoing MEASURE study that describes the relationship between post-HCT genomic MRD emergence and overt relapse will provide valuable insights into whether such a pre-emptive strategy is feasible. MEASURE will also inform genomic subgroups that may benefit from maintenance in the absence of peri-HCT MRD. Future research in this space should leverage data from this study and others to identify specific populations that benefit from post-HCT maintenance, and those that may be spared further therapy, tailoring interventions to individual patient needs.

## Conclusions & recommendations

- **Upfront Transplantation for ELN Adverse-Risk Disease:** Patients with ELN Adverse-Risk disease have a high relapse risk and often do not achieve MRD negativity. We recommend upfront allogeneic HCT for all fit patients in CR, regardless of MRD status. Post-HCT relapse mitigation strategies, ideally within clinical trials, should be considered, especially for those not receiving intensive conditioning.
- **ELN Favorable-Risk Disease with Persistent or Recurrent MRD:** For patients with persistent or recurrent MRD after induction, individualized treatment is crucial. Allo-HCT is a viable option. If MRD negativity is achieved after additional therapy, particularly in borderline-fit patients, a watchful waiting approach with serial MRD monitoring may be appropriate. Some lower-risk genomic profiles may allow for less aggressive intervention.
- **ELN Intermediate-Risk Disease:** Allogeneic HCT is recommended for all suitable patients in CR1, regardless of MRD status. For MRD-negative, borderline-fit patients, watchful waiting with serial MRD assessment may be an alternative. Additional therapy to achieve MRD negativity in MRD-positive patients should be considered case-by-case.
- **Pretransplant Eradication of MRD:** Achieving MRD negativity before HCT is linked to better outcomes but must be balanced against the risks of delaying HCT. Outcomes for patients who achieve MRD negativity after post-induction therapy are comparable to those MRD-negative after induction. For certain high-risk cytogenetic and molecular profiles, achieving MRD negativity is challenging, even with further consolidation or salvage therapies, emphasizing the need for careful patient selection and timely decision-making.
- **Tailoring Conditioning Therapy to MRD Status:** For fit MRD-positive patients, we recommend MAC due to its association with lower relapse rates. For less fit patients, it is crucial to balance relapse prevention with transplant toxicity. Intensified RIC and novel approaches may benefit high-risk patients not eligible for MAC. Additionally, MRD-directed therapies could be considered for those unfit for intensive conditioning, although standardized guidelines are lacking. Persistent molecular disease, especially in cases with *NPM1* and *FLT3*-ITD mutations, may necessitate further therapy to eradicate MRD before alloHCT. A large, genomically annotated prospective study is needed to refine these treatment strategies.
- **MRD Assessment in Clinical Practice:** MRD assessment is evolving, with technologies like NGS offering enhanced sensitivity. The Pre-MEASURE study underscores the potential of error-corrected NGS, though issues of timing, methodology, and standardization remain. MRD results should be integrated into clinical decisions with caution, combining various techniques and clinical judgment.
